# Precision Medicine: Disease Subtyping and Tailored Treatment

**DOI:** 10.3390/cancers15153837

**Published:** 2023-07-28

**Authors:** Richard C. Wang, Zhixiang Wang

**Affiliations:** 1 Department of Radiology and Radiological Sciences, Johns Hopkins University School of Medicine, Baltimore, MD 21287, USA; rwang93@jhmi.edu; 2 Department of Medical Genetics, Faculty of Medicine and Dentistry, College of Health Sciences, University of Alberta, Edmonton, AB T6J 5H4, Canada

**Keywords:** precision medicine, subtyping, biomarkers, multiomics, tailored treatment, targeted therapy, pharmaco-omics, functional precision medicine, electronic health record, big data analytics

## Abstract

**Simple Summary:**

The genomics-based concept of precision medicine began to emerge following the completion of the Human Genome Project. In contrast to evidence-based medicine, precision medicine will allow doctors and scientists to tailor the treatment of different subpopulations of patients who differ in their susceptibility to specific diseases or responsiveness to specific therapies. In this review, we examine the history, development, and future perspective of precision medicine. We also discuss the concepts, principles, tools, and applications of precision medicine and related fields.

**Abstract:**

The genomics-based concept of precision medicine began to emerge following the completion of the Human Genome Project. In contrast to evidence-based medicine, precision medicine will allow doctors and scientists to tailor the treatment of different subpopulations of patients who differ in their susceptibility to specific diseases or responsiveness to specific therapies. The current precision medicine model was proposed to precisely classify patients into subgroups sharing a common biological basis of diseases for more effective tailored treatment to achieve improved outcomes. Precision medicine has become a term that symbolizes the new age of medicine. In this review, we examine the history, development, and future perspective of precision medicine. We also discuss the concepts, principles, tools, and applications of precision medicine and related fields. In our view, for precision medicine to work, two essential objectives need to be achieved. First, diseases need to be classified into various subtypes. Second, targeted therapies must be available for each specific disease subtype. Therefore, we focused this review on the progress in meeting these two objectives.

## 1. Introduction

In 2011, The genomics-based concept of precision medicine began to emerge following the completion of the Human Genome Project. The US National Research Council defines precision medicine as “an emerging approach for disease treatment and prevention that takes into account individual variability in genes, environment, and lifestyle for each person”. This approach is in contrast to a one-size-fits-all approach and will allow doctors and scientists to tailor treatment to different subpopulations of people based on their unique disease susceptibility and/or treatment response.

Following the national Precision Medicine Initiative in the United States launched by then President Obama in 2015, precision medicine also received public attention. The number of worldwide Google searches for the term “precision medicine” dramatically increased, even surpassing the number of searches for “personalized medicine” [1]. By 19 February 2019, it was reported that “precision medicine” had been searched 5.5 million times vs. the 5.2 million for “personalized medicine” [2]. With rapid development, precision medicine has become a term that symbolizes the most advanced modern and future medicine. So, then, what exactly is precision medicine? What are its principles, tools, and applications? What has precision medicine achieved so far, and what is its future promise? And what is its relationship with other medical concepts like personalized medicine, P4 medicine, individualized medicine, and stratified medicine? This review aims to answer all these questions. We examine the history, development, and future perspective of precision medicine. We also discuss the concepts, principles, tools, and applications of precision medicine and related fields. In our view, for precision medicine to work, two essential objectives need to be achieved. First, diseases need to be classified to various subtypes. Second, targeted therapies must be available for each specific disease subtype. Therefore, we focused this review on the progress in meeting these two objectives.

## 2. The Evolving of Medicine Concepts and the Emerging of Precision Medicine

**Figure 1:**
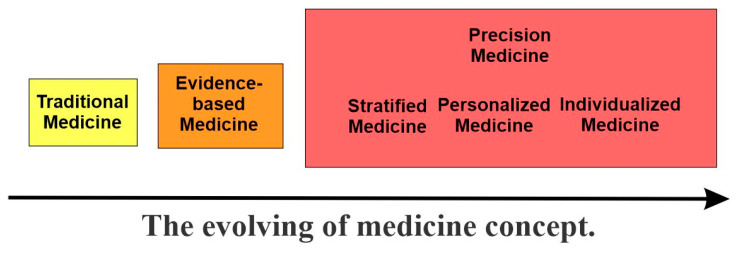
The evolution of the medicine concept.

Often, the term “precision medicine” is used synonymously with similar terms like personalized medicine, stratified medicine, individualized medicine, tailored medicine, and P4 medicine. All these terms were recently developed to contrast the traditionally used “evidence-based medicine”, which in turn is the advancement from “traditional medicine”. In this section, we will briefly describe all these terms and their relationship with precision medicine (Figure 1).

### 2.1. Traditional Medicine and Evidence-Based Medicine

According to the World Health Organization, the conscientious, explicit, and judicious use of traditional medicine is “the sum total of the medical knowledge, skills, and practices based on the theories, beliefs, and experiences indigenous to different cultures”. Its basis in culture-specific beliefs and experiences is key, and it often reflects a more holistic approach to medical care. Prominent examples of traditional medicine include the use of herbal medicine in China or the practice of Ayurveda in India. These approaches are not necessarily supported on a scientific basis but are rather a result of knowledge and practices passed down through generations within a particular culture.

While traditional medicine is rooted in cultural and historical practices, evidence-based medicine (EBM) is grounded in the scientific process. In EBM, the current best evidence is identified and explicitly used in decision making regarding the care of individual patients. In this way, individual clinical expertise will be integrated with the best available external clinical evidence through systematic research [3,4].

The key difference between traditional medicine and EBM is not whether they consider evidence when guiding clinical care (as they both do) but rather the type of evidence needed. Traditional medicine typically relies on anecdotal evidence, while EBM aims for critically appraised scientific evidence that generally requires statistical significance. There has been an overall shift from traditional medicine toward EBM, especially with the onset of new methods for obtaining scientific evidence, such as randomized-control trials. One of the biggest successes of EBM is the development of systematic reviews and meta-analyses. By using these methods, researchers can identify multiple studies on a specific topic and then critically analyze them to identify the best available evidence [5].

In EBM, data are collected from large cohorts, from which mean values or figures are derived; the results are then used to infer clinical recommendations to the entire population. Outliers are frequently removed in the analysis or unacknowledged in the resulting recommendations. This approximates the “one size fits all” approach to medical care. Most people will fall within the mean estimates of EBM for any given trait; however, for one or several conditions there will always be some individuals who are outliers. These outliers patients will not respond to the recommended evidence-based medical practices. In these cases, EBM is not good enough to meet the needs of these patients.

In this sense, EBM stands at odds with precision medicine, which focuses on collecting a massive number of data-points for the individual rather than the population. As medical practice has increasingly shifted from generic (“one size fits all”) to precise, there will be a need to reconcile evidence-based medicine and precision medicine. Despite their differences, they can and should be viewed as mutually complementary [6,7].

### 2.2. The Emergence of Precision Medicine

#### 2.2.1. Overview

The term precision medicine may be relatively new, but its concept has existed for a long time. Hippocrates (460-370 BCE) once famously said that “It’s far more important to know what person the disease has than what disease the person has” [2,8]. There are numerous medical practices in existence that share the concept of precision medicine. One great example is blood typing for blood transfusions.

However, for most of medicine’s history, EBM has been adopted by physicians to treat their patients, simply because they do not have a better choice [9].

Fortunately, due to the recent development of a large-scale biological data base and computational tools used to analyze the large sets of data, a more personalized and precise approach to medicine is becoming possible.

The origin of the term “precision medicine” is not completely clear, but it is believed to have been first introduced in 1979 by Canadian researcher Dr. L.Y. Wei. He published an article entitled “Scientific advances in acupuncture” in the first issue of volume 7 of the American Journal of Chinese Medicine [10]. In the article, he introduced the “three P’s” of medicine: preventative medicine, precision medicine, and people medicine. Wei described the “three P’s” with a narrow focus on Acupuncture. In the absence of current-day genomic data and tools, Wei imagined an image-based approach to achieve these three Ps [10].

The genomics-based concept of precision medicine, as defined by the US National Research Council in 2011 [11], began to emerge following the completion of the Human Genome Project. It is expected that knowledge regarding the genetic basis of diseases will lead to better tailored treatments.

#### 2.2.2. Stratified Medicine

According to The Academy of Medical Sciences of the UK, stratified medicine is the grouping of patients based on risk of disease or response to therapy by using diagnostic tests or techniques. With stratified approaches to medicine, we can greatly increase the certainty of clinical decision making by using our improved knowledge of diseases and diagnoses [12]. Stratified medicine aims at matching a therapy with a specific patient population through the use of clinical biomarkers. In this way, patients could maximize the benefit of the treatments [2,13].

#### 2.2.3. Personalized Medicine

Personalized medicine, like precision medicine, emphasizes the importance of tailoring therapeutic approaches to the specific needs and characteristics of each patient. While they are often used interchangeably, there are slight differences in the meaning and connotation of each term, which resulted in the former falling out of favor.

Personalized medicine was first used by a Canadian physician in a 1971 publication, where he described the concept of treating a patient as a person rather than a condition [14]. However, the current-day concept of personalized medicine only emerged at the end of the last century with the development of the Human Genome Project. This new concept was introduced to the public in an article entitled “New Era of Personalized Medicine: Targeting Drugs for Each Unique Genetic Profile” in The Wall Street Journal in April 1999. A few months later, the article was reprinted in The Oncologist [15,16]. Under this new concept, personalized medicine was defined as an emerging practice of medicine that used an individual’s genetic profile to guide decisions regarding the prevention, diagnosis, and treatment of disease.

Despite its widespread use, however, there appeared to be considerable confusion in the public about what “personalized medicine” really meant. For instance, a 2013 survey found that only 4% of the public understood what the term was intended to mean [17]. One key concern from the US National Research Council was that “personalized medicine” suggested treatment or preventive measures that were developed uniquely for each individual patient, which is not the case. This led to the rise of the term “precision medicine”, made prominent by the National Academy of Sciences in 2011. Under this new term, it is clearer that therapies are being developed uniquely for each subgroup of patients with a similar response to a treatment, rather than an individual patient [1].

#### 2.2.4. Individualized Medicine

While the term individualized medicine [18] and related terms such as individualized care [19] and individualized therapy [20] have been around for many years, Topol is the one who proposed to replace the widely used term personalized medicine with individualized medicine in 2014. Based on a recent survey that found that only 4% of the public understand what “personalized medicine” is intended to mean [17], he argued that personalized medicine has given rise to considerable confusion. He believed that “individualized medicine” is a better term, since it relates not only to medicine that is particularized to a human being but also to the future impact of digital technology on individual’s driving their health care [17].

However, the term individualized medicine had been used for many years by others to mean “truly” individualized [2]. Individualized medicines have been used to mean the matching of an individual patient with a specifically tailored therapy that varies inherently for each patient. Recent advances in medicine make this increasingly possible. The best example is the cancer vaccine Oncophage, a therapy for earlier-stage kidney cancer. Oncophage was approved in Russia in 2008. It was also granted fast track and orphan drug designations from the US Food and Drug Administration. For this therapy, one vaccine is only suitable for a specific patient as the vaccine is produced using the tumor cells taken from this specific patient during surgery. Other examples include some stem-cell-based therapies [13].

#### 2.2.5. P4 Medicine

P4 medicine stands for predictive, preventive, personalized, and participatory medicine, which was proposed by Hood in 2004 [21,22]. By using a large network of information on health and disease, P4 medicine aims to enable a comprehensive medical approach to develop public health intervention and monitor the health status of the population.

## 3. Central Tenets and Two Essential Objectives of Precision Medicine

Precision medicine has often been described as the “right drugs for the right patients at the right time”. To achieve this goal, the current “precision medicine” model is proposed to precisely classify patients into subgroups sharing a common biological basis of diseases for more effective treatment and improved care outcomes [23].

**Figure 2:**
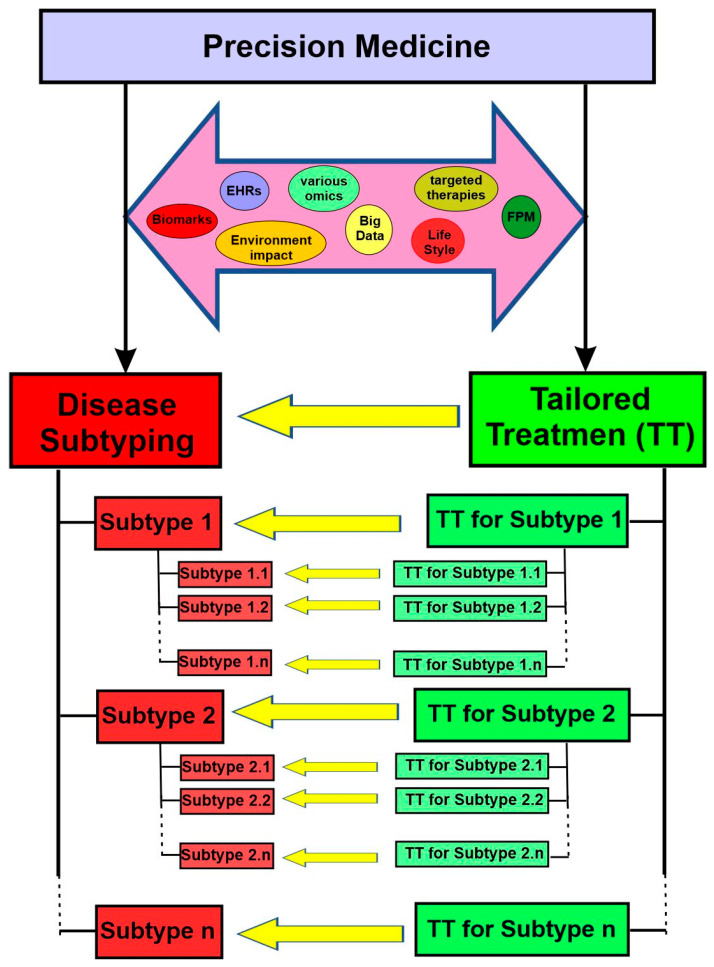
Central tenets and objectives of precision medicine. The current “precision medicine” model is proposed to precisely classify patients into subgroups sharing a common biological basis of diseases for more effective tailored treatment (TT) to achieve improved outcomes. With rapid progress in various omics, targeted therapies, functional precision medicine (FPM) models, comprehensive electron health records (EHRs), and big data analytics in the last two decades, we are able to divide many common diseases into subtypes and sub-subtypes and are able to develop tailored medicine for many of those sub-subtypes. With continued efforts, we will achieve more accurate and precise treatment for individual patients.

For precision medicine to work, two essential objectives need to be achieved. First, the disease needs to be classified into various subtypes. Second, there must be targeted therapies available for each specific subtype. On this basis, patients can be subgrouped into different subtypes and then treated with the appropriate, tailored therapies (Figure 2). With rapid progress in various omics, targeted therapies, functional precision medicine models, comprehensive electron health records (EHRs), and big data analytics in the last two decades, both objectives are within reach now.

**Figure 3:**
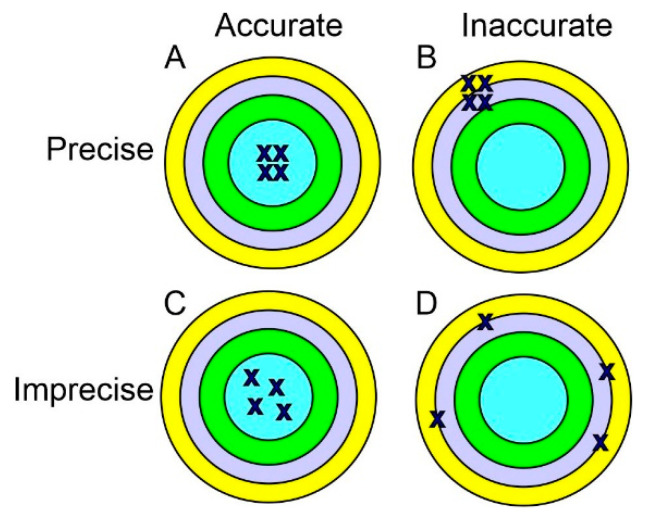
Precision medicine, as defined, explicitly includes the concepts of both precision and accuracy. (**A**) Precise and accurate, the goal of precision medicine. (**B**) Precise but inaccurate. (**C**) Accurate but imprecise. (**D**) Inaccurate and imprecise.

While accuracy does not appear in its name, precision medicine explicitly includes the concepts of both accuracy and precision. Accuracy reflects the degree to which the result of a given measurement conforms to the correct value or a standard, whereas precision refers to how close the repeated measurements are to each other. This can be figuratively explained using target shooting. Accurate but imprecise shots scatter widely around the center, whereas precise but inaccurate shots cluster together away from the center (Figure 3). Both accuracy and precision are essential for the success of precision medicine. For example, genomes should be faithfully represented (accuracy) and the genetic tests should be reproducible (precision). If a genetic test is imprecise and/or inaccurate, it will cause serious consequences for the individuals and families involved.

## 4. Disease Subtyping

### 4.1. Overview

As an essential objective of precision medicine, disease subtyping is the task of classifying a disease into distinct patient subgroups based on specific patient characteristics, which can then guide treatment decisions based on which subgroup a patient belongs to [24]. More research with different approaches has been conducted to identify homogeneous patient subgroups. Both quantitative models based on multiple high-throughput data and qualitative models based on clinical observations are actively explored. The subtyping of cancer [25,26], autism [27], asthma [28], autoimmune diseases [29,30], cardiovascular diseases [31], and Parkinson’s disease [32] are good examples in this regard

Disease subtyping can benefit both the science and practice of medicine. One area is to make a treatment decision based on the prognosis of the other patients in the same disease subtype. For example, if an individual’s prognosis points to a rapidly worsening condition without treatment, a treatment even with strong side effects could be well justified. Moreover, subtypes can also help predict the expected costs of the treatment. Different subtypes may have treatments that differ in their cost to both the patient and the healthcare system.

Scientifically, subtyping can guide and help the design of genetic testing to identify the molecular determinants that differ between subgroups. Disease subtyping can also improve clinical trials for complex diseases with tremendous heterogeneity by enabling targeted recruitment. Such analyses can allow clinical scientists to better understand the causes of related diseases.

Of note, these subtypes are also called endotypes if they have been established to be causally associated with the underlying mechanism of disease [33]. Additionally, the “verotype” (vero means “true” in Latin) is described by Boland and colleagues to represent the true population of similar patients for treatment purposes [34].

### 4.2. Approaches for Disease Subtyping in Precision Medicine

Although disease subtyping has been conducted for many years, it was conducted as a by-product of clinical experience in past times. When clinicians noticed the presence of outlier patients with different symptoms or responses to treatments, they followed up with this observation by retrospectively or prospectively performing more research to confirm their observation. However, this type of subtyping was limited by the expertise and resources of individual doctors. Things changed in the last two decades. With the rapid development of modern high-throughput biotechnologies, we can now measure differences between individuals at the cellular and molecular levels with various “-omics”. The cost of measuring these “-omic” data (such as genomics, proteomics, and metabolomics data) has decreased significantly, which allows the collection of such data on a large number of patients. While the cost of sequencing the first human genome two decades ago was USD 400 million, the cost to do so was approximately USD 1500 in 2015 [35] and close to USD 200 today. With these enormous new data, scientists have shifted their focus toward computationally driven approaches to subtyping.

**Figure 4:**
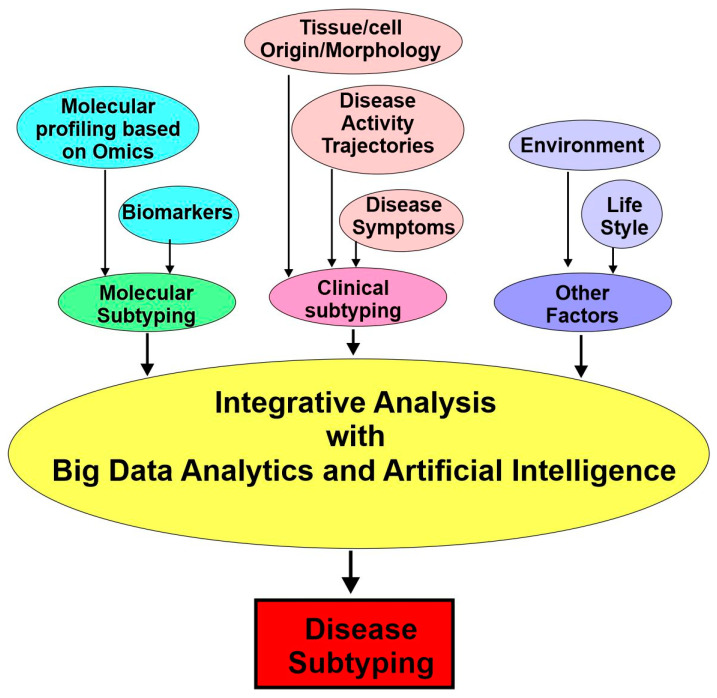
Disease subtyping. Diseases can be subtyped using various approaches, including molecular subtyping with various biomarkers and data from diverse omics; clinical subtyping based on EHRs; and environmental, social, and behavioral factors. In precision medicine, all these diverse data surrounding an individual’s health will be carefully integrated and analyzed by big data analytics and artificial intelligence to achieve the final subtyping.

These computational approaches include (1) molecular subtyping with various biomarkers and data from diverse -omics and (2) clinical subtyping based on electronic health records (EHRs) and deep phenotyping. Finally, the diverse data surrounding an individual’s health will be carefully integrated and analyzed further by considering environmental, social, and behavioral factors (Figure 4).

### 4.3. Molecular Subtyping: Biomarkers

Without the clinical application of various omics, the complete molecular profiling of patients is impossible. However, biomarkers have been widely used for disease subtyping and have made significant contributions to the progress of precision medicine. It is argued that the success of precision medicine is largely predicated on the identification of highly predictive markers of efficacy [36,37].

**Figure 5:**
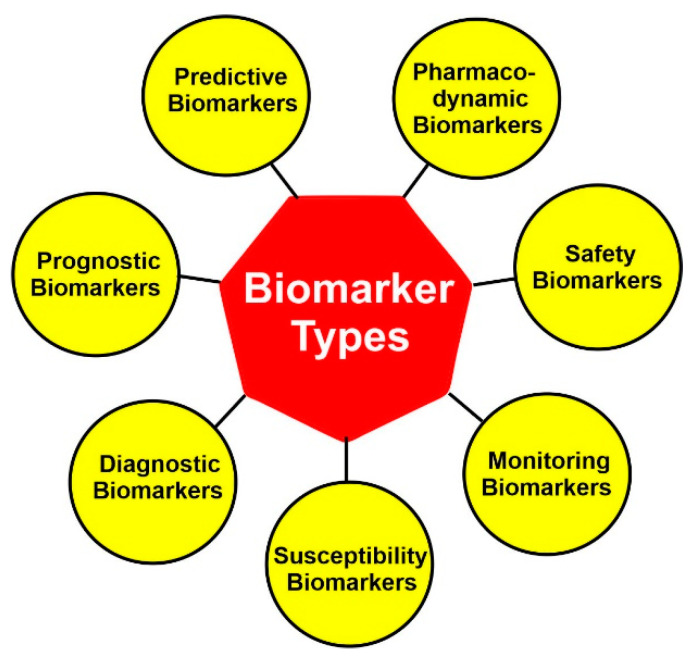
Various types of biomarkers.

According to FDA, biomarkers are classified into seven categories: diagnostic biomarkers, prognostic biomarkers, predictive biomarkers, susceptibility biomarkers, monitoring biomarkers, safety biomarkers, and pharmacodynamic biomarkers (Figure 5). Among them, the diagnostic, prognostic, and predictive biomarkers are the ones primarily used for precision medicine.

#### 4.3.1. Diagnostic Biomarkers

Diagnostic biomarkers are markers for the detection and/or confirmation of the presence of a disease. They are also used to identify individuals with a subtype of the disease. Ideally, diagnostic biomarkers can detect diseases before they become symptomatic. For example, serum prostate-specific antigen (PSA) is used for the early detection of prostate cancer [38]. The HER2 expression level has been used to subtype HER2-positive breast cancer [39,40]. In addition, a diagnostic biomarker may also be used to define disease endotypes and guide the choice of a specific treatment if it reflects the molecular pathology of the disease. For instance, HER2-positive breast cancer is treated by trastuzumab as a targeted therapy.

#### 4.3.2. Prognostic Biomarkers

A prognostic biomarker projects the disease trajectory, such as the likelihood of progression, remission, and future clinical events, independent of treatment [2,41]. A good example is the PIK3CA mutation status in HER2-positive metastatic breast cancer. If a patient contains a PIK3CA mutation in tumor cells, she has worse progression-free survival compared with a patient without the mutation [42]. In this case, the PIK3CA mutation status is a prognostic biomarker. The presence of this prognostic biomarker reflects the disease’s underlying biology and natural history, and the identification of this biomarker may also inform potential treatment strategies. Another example is the different prognosis between different subtypes of breast cancer. It is well known that the subgroup of triple-negative early-stage breast cancer has a worse survival outcome than the subgroups with hormone receptors—and/or HER2-positive disease [43,44].

#### 4.3.3. Predictive Biomarkers

A predictive biomarker is used to differentiate the treatment effect between patients in different disease subtypes or between patients and normal people. The difference in benefits could be qualitative or quantitative. Thus, predictive biomarkers are vital for guiding precision medicine because they have the potential to identify individuals that are more or less likely to respond to a given treatment [2,41]. For example, patients with immune-enriched tumors (the expression of any 9 or more of 14 immune function genes) seem to derive benefit from trastuzumab, whereas those with non-immune-enriched tumors do not seem to derive benefits [45]. Thus, this subset of immune function genes could serve as a predictive biomarker for the response to trastuzumab in these patients.

#### 4.3.4. Other Biomarkers

Pharmacodynamic biomarkers are response biomarkers that indicate drug effects on the target in an organism. They can be used to examine the link between drug regimens, target effects, and biological tumor responses.

A susceptibility biomarker is a biomarker that indicates the likelihood that an individual who does not currently have a clinically apparent disease or medical condition will develop a disease or medical condition.

Monitoring biomarkers are used to assess the status of a disease or medical condition. To be effective, the monitoring biomarkers need to be measured repeatedly. Monitoring biomarkers are also useful in assessing the evidence of exposure to a medical product or an environmental agent.

Safety biomarkers reflect the potential or presence of toxicity related to a therapeutic agent or an environmental factor. Safety biomarkers are measured before or after the exposure.

#### 4.3.5. Biomarker Combinations

Because the prognosis and treatment outcomes of a patients are determined by the combination of multiple factors, including genetic, epigenetic, phenotypic, and environmental factors, a single biomarker can only capture a part of all these aspects and has a limited ability to predict a patient’s response to a specific treatment. Thus, a combination of multiple biomarkers provides a better indicator for patient subtyping and treatment decision making. It is a sophisticated task to identify the optimal linear and nonlinear biomarker combinations that offer the highest sensitivity and specificity for a given classification. This task is now accomplished by machine learning and multivariate statistical analysis.

The study of biomarker combinations is still in its early stage with the combinations of biomarkers of the same layer, for example, several metabolomic markers or protein markers. However, with advancements in omics technologies, bioinformatics, and computational tools, future biomarker combinations aim to integrate these various orthogonal (independent) biologic approaches.

### 4.4. Molecular Subtyping: -Omics

**Figure 6:**
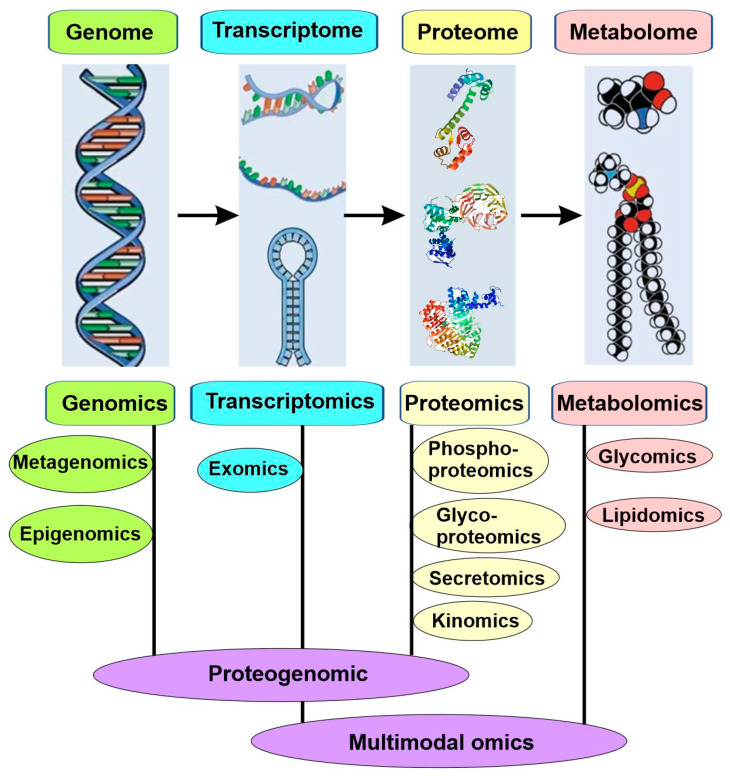
Various types of omics.

Most common and life-threatening diseases are multifactorial, such as cancers, cardiovascular diseases, and diabetes. Due to their complexity and heterogeneity, both their existing biomarkers and the traditional approaches used to identify new biomarkers are insufficient for precision medicine. Consequently, the new approach for the molecular subtyping of diseases is based on big data collected with multiple omics (Figure 6).

High-throughput technologies have revolutionized medical research since the completion of the Human Genome Project at the beginning of the millennium. These new technologies can generate a high-throughput and high-resolution snapshot of any biological system of interest, which constitutes “Omics”. Omics is a nomenclature broadly used to describe a collective study of molecular characterization and the quantification of biological molecules via high-throughput technology. Based on the subdomains the molecules belong to, these omics were described as genomics, epigenomics, transcriptomics, proteomics, and metabolomics, among others (Figure 6).

The most relied-upon high-throughput technologies in omics is next-generation sequencing (NGS), which sequences millions of individual DNA fragments simultaneously to generate a massive amount of molecular data with greater efficacy, speed, and cost-effectiveness. NGS has benefited almost all subdomains of omics research.

Generation and analyses of high-throughput molecular data through various omics allow the unbiased biomedical discovery of disease subtypes via the unsupervised clustering of either individual or multiple sources of molecular data [24].

#### 4.4.1. Genomics

Genomics focuses on the study of entire genomes and is the first omics discipline to appear. This is different from “genetics”, which only studies individual variants or single genes. Genomic studies interrogate genetic variations, contributing to both mendelian and complex diseases. Important approaches in genomics include genome-wide association studies (GWASs), whole-genome sequencing (WGS), and whole-exome sequencing (WES).

GWASs have been successively used to identify thousands of genetic variants associated with complex diseases in various human populations. In GWASs, more than a million genetic markers are analyzed for a large group of individuals. Any differences in minor allele frequencies (such as single nucleotide polymorphisms or SNPs), if statistically significant between patients and controls, are treated as the evidence of association. GWASs have contributed significantly to our understanding of complex diseases.

Whereas GWASs aim to identify associations between genetic variants and diseases, WGS and WES focus on sequencing the genome. WGS aims to sequence the complete genome of an individual, including both coding and noncoding regions, while WES focuses specifically on sequencing the exome or the coding segments of the genome actually transcribed. Both WGS and WES rely on NGS to generate the vast amounts of data needed. When compared with WGS, WES can be performed at a faster rate (hours vs. days), deeper coverage (>100× compared to 30×), and lower price because the exome only comprises 1.5% (40 Mb) of our genome. However, WGS can yield much more information than WES. It is known that 80% of the loci involved in complex diseases are located in the noncoding regions of the genome, which comprise 98.5% of the genome and play important regulatory roles. With the continuing drop of the WGS price, it is more cost-efficient to perform WGS for genotyping, pharmacogenomics, genome-wide association studies (GWASs), and single-nucleotide polymorphism (SNP) analysis. Microarray or bead-based SNP analysis is also a viable low-cost choice, although with considerably lower coverage than WGS.

Another powerful genomics tool to reveal cellular complexity is single-cell genomic sequencing (SGS), which focuses on analyzing the genome of individual cells. This is highly useful for exploring individual genomic variation (mosaicism), such as when differentiating between tumor and nontumor cells.

#### 4.4.2. Epigenomics

Although the genome that we are born with is more or less constant, it undergoes chemical modifications during our lifetime. Two of the most common and well-studied chemical modifications are DNA methylation and histone acetylation. The sum total of these modifications is referred to as the epigenome, and the quantification and characterization of the epigenome is called epigenomics. Covalent modifications of DNA and histones play an important role in gene transcription. Epigenic modifications are regulated both by genetic and environmental factors and are sometimes heritable and long lasting. Many epigenome-wide association studies have revealed the important role of epigenetic modifications in biological processes and disease development. For example, differentially methylated regions of DNA can be used as indicators of disease status for cancer, cardiovascular disease, metabolic syndrome, and many other pathophysiologic states. Epigenetic signatures are often tissue-specific. In addition to DNA methylation and histone acetylation, MicroRNA (miRNA), which can either interfere in translation or destroy coding mRNA, can also regulate gene expression and thus also forms a part of epigenomics. miRNAs are potential diagnostic, prognostic, and predictive biomarkers in diabetes, cancer, diabetes, and other diseases [26,46].

As the bisulfate treatment of DNA converts nonmethylated C’s to U’s without affecting methylated C’s, it is commonly used as the first step before microarrays or WGS to assess DNA methylation. Genome-wide histone acetylation is determined by the combination of several procedures, including chromatin immunoprecipitation (ChIP), the binding of histone modification-specific antibodies to DNA, and ChIP-seq by NGS. Microarrays, qRT-PCR, and small RNA-seq are the techniques used to identify the relevant miRNAs [2]. All these techniques have resulted in the successful mapping of several human epigenomes, which have helped us identify epigenetic biomarkers and develop epigenetic-specific therapeutics (i.e., histone deacetylase inhibitors).

#### 4.4.3. Transcriptomics

Transcriptomics was initiated in the early 1990s and is the study of the transcriptome using high-throughput methods [47]. The transcriptome is the complete set of RNA transcripts produced by the genome in a specific cell or under specific conditions. Microarrays have been the main technique used to analyze transcription for many years since the late 1990s; however, today, RNA-seq is the method of choice for transcriptomic profiling due to the advancement of NGS technology and a lowering of its price [2].

Transcriptomics can reveal details of an organism’s biology by obtaining information regarding the gene expression in different tissues, conditions, or time points. It can also reveal the changes of gene expression in different organisms. In addition, it helps to understand the functions of previously unannotated genes. Transcriptomics has contributed significantly to our understanding of human disease [47].

However, what is measured by transcriptomics is only a snapshot of the transcriptome, which varies extensively with time, space, and conditions. Therefore, when it is used to compare transcriptomic profiles of different biologic samples, it is very importance to standardize the experimental conditions. The recently developed spatial transcriptomics helps achieve this [48]. With spatial transcriptomics, the researchers are able to spatially localize and quantify gene expression within tissues and cells that are in their native state. Spatial transcriptomics can be used to generate transcriptome maps and atlases based on unique gene expression profiles and connect them to their cellular location and morphology. Spatial transcriptomics can also quantify differences in gene expression among cells and help to gain insights into diseases’ causality [48].

#### 4.4.4. Proteomics

Proteomics is the method used by researchers to study the composition, function, interactions, and structures of proteins [2]. Proteomics is achieved through the analysis and quantification of proteins in cells or body fluids via MS-based high-throughput methods. Proteomics provides a better understanding of the structure and function of the organism than genomics because protein is the molecule to carry out the cell functions. Not all mRNA is translated into protein; therefore, gene expression levels are only partially reflecting the corresponding protein levels. Moreover, post-translational modifications, such as phosphorylation, lipidation, and ubiquitination, are important determinants of protein function [2,49].

There are three major types of proteomics: expression proteomics, structural proteomics, and functional proteomics. Expression proteomics is the best-known and most-used type of proteomics. It studies the expression of protein both quantitatively and qualitatively. It can also reveal the post-translational modification by performing specific proteomics. For example, phosphoproteomics can reveal the phosphorylation status of the proteins under specific conditions. In general, expression proteomics is used to determine the difference in protein expression between two conditions, such as patients and controls. It can also discover disease-specific proteins and new proteins involved in signaling pathways. Structural proteomics employ X-ray crystallography and nuclear magnetic resonance spectroscopy to reveal the three-dimensional structure of functional proteins. A good example of structural proteomics is the study of the nuclear pore complex. Functional proteomics aims to reveal the protein functions and the underly molecular mechanisms. It also determines the interaction of a protein with an unknown binding partner in a specific protein complex involved in a specific signaling process. Functional proteomics has the power to identify the biological function of the protein. Furthermore, the determination of protein–protein interactions in vivo can lead to the understanding of the sophisticated cellular signaling pathways [49].

#### 4.4.5. Metabolomics

Metabolomics is the method to study small molecules, commonly known as metabolites, within cells, biofluids, tissues, or organisms on a large scale. Collectively, the metabolites and their interactions within a biological system are known as the metabolome [2,50,51]. The major classes of metabolites include amino acids, carbohydrates, nucleotides, lipids, coenzymes, and cofactors. The analysis of these diverse types of metabolites requires different platforms, such as ultra-performance liquid chromatography (UPLC), nuclear magnetic resonance (NMR), and mass spectrometry (MS) [52],

Metabolomic abnormalities have been associated with many human diseases, including cardiovascular disease, diabetes, and cancer. With the development of analytical technologies, metabolomics is now a powerful tool for elucidating the pathological mechanisms of diseases, discovering novel drug targets, interpreting the mechanisms of drug action, predicting drug responses, identifying diagnostic biomarkers, and enabling the precision treatment of patients [52].

Metabolomics simultaneously quantifies multiple small molecule types, such as amino acids, lipids (lipidomics), carbohydrates (glycomics), or other products of cellular metabolic functions, by using multiple high-throughput analytical platforms, including mass spectrometry (MS), nuclear magnetic resonance (NMR), and ultra-performance liquid chromatography (UPLC) [52] (Figure 6). Metabolite levels and relative ratios are reliable parameters of metabolic function, and the deviation from the normal range often indicates the disease. Through the quantitative measures of the metabolite levels, metabolomics enables the discovery of novel genetic loci that regulate small molecules in body liquids and various tissues.

#### 4.4.6. Microbiomics and Metagenomics

The microbiome is the complete load of microorganisms present in a human body. Microbiomics is defined as the characterization of the molecules responsible for the structure, function, and dynamics of the microbiome. The human gut, skin, and mucosal surfaces are all colonized by microorganisms, including bacteria, viruses, and fungi; these microorganisms and their environment are collectively known as the microbiota. The collection of all the genes of the microbiota is known as the microbiome. The microbiome alters the metabolome and epigenome of a person, thereby having significant implications in precision medicine [53,54]. In particular, the human gut contains more than 100 trillion microorganisms from more than 1000 different species and these microorganisms play an important role in a person’s metabolism, nutrition, and immune function [2]. The microbiome is also implicated in various diseases and drug interactions. For example, digoxin is inactivated by specific gut bacteria. The microbiota composition varies substantially between individuals and between populations due to many factors, including seed during birth, age, diet, drugs, etc. Perturbations in gut bacteria have been implicated in various disorders, including autism, obesity, diabetes, cancer, colitis, and heart disease.

Two methods are commonly used to profile the microbiome. The first method is to amplify and then sequence certain hypervariable regions of the bacterial 16S rRNA genes followed by clustering the sequences into operational taxonomic units. The second method is to sequence the total DNA using shotgun metagenomics sequencing, which provides additional resolution to distinguish genetically close microbial species.

Another term closely related to microbiomics is metagenomics. Metagenomics is defined as the study of the genomes of whole biological communities from a particular habitat. It is often used to study microorganisms, such as those residing on the human gut or skin. Therefore, metagenomics is sometimes considered another name for microbiomics [55].

#### 4.4.7. Proteogenomics and Multimodal Omics

Although omics data of each type are useful as biomarkers of the disease process, as well as provide a list of differences associated with the disease, they are limited to correlations rather than revealing the causative relationship. With the increase in the ability to identify genetic variants associated with complex diseases, we now realize that the genes identified so far only explain a small portion of the heritable component for specific diseases. Moreover, common diseases usually result from changes in gene regulation rather than the coding regions of genes as in Mendelian diseases. Moreover, we also understand that the same genetic variations usually cause different final outcomes due to differences in environmental and genetic backgrounds. Therefore, it is essential to integrate different omics data types to identify molecular patterns associated with diseases, elucidate potential causative changes that lead to diseases, and determine treatment targets that can then be tested in further molecular studies [56].

The outcome of the first such integration is the emergence of proteogenomics as an independent field in 2004 [57]. Proteogenomics is at the interface between genomics and proteomics (Figure 6). Through proteogenomics, scientists can identify novel peptides that are not present in reference protein sequence databases but present in protein sequence databases generated from genomics and transcriptomics; in addition, the data from proteomics can also be used to provide protein-level evidence of gene expression and to help refine gene models [56].

Early efforts in proteogenomics were hindered by the low sensitivity of proteomic technology, which cannot support proteogenomics. However, the great improvements in mass spectrometry, including protein separation and enrichment methods, new instrument types, alternative fragmentation mechanisms, and advanced data-acquisition strategies, substantially increased the depth of protein detection, which allows its match with that of genomics and transcriptomics [58,59]. With additional progress in top-down proteomics—a technology useful for proteogenomic characterization—proteogenomics became possible and was rapidly adopted [60].

Proteogenomics has resulted in several advancements in omics research. It showed that RNA expression levels are often poor predictors of actual protein levels, which inspires the thorough characterization of signaling and regulatory pathways to determine the pathways’ activation under given conditions. Proteogenomics also facilitated customized proteomics database searching to identify novel proteins and genomic aberrations that potentially act as the driver of the disease. In the context of precision medicine, proteogenomics can help provide a molecular diagnosis of a patient’s disease and successfully attribute diseases to specific molecular subtypes. Patients in each subtype share pathogenic mechanisms and respond similarly to targeted therapies. Proteogenomics will allow better recommendations for treatment regimens that are most likely to be beneficial to patients.

Following the successful integration of genomics, transcriptomics, and proteomics to form proteogenomics, other omics, such as epigenomics, have also been integrated. Since 2019, the approach of integrating various omics has been more commonly referred to as multimodal omics or multiomics [61,62]. Through multiomics, metabolomics scientists can have a greater understanding of the flow of information, from the cause of disease (genetic, developmental, or environmental) to relevant interactions, as well as the functional consequences [63]. Multiomics approaches can be divided into three categories, “genome first”, “phenotype first”, and “environment first”. The “genome first” approach seeks to determine the mechanisms by which gene loci contribute to the disease. The “phenotype first” approach focuses on the pathways contributing to the disease. The “environment first” approach examines how the environment interacts with genetic variation and affects pathways.

More recently, technological advances in single-cell analysis have enabled multimodal omics measurements in a single cell, whether these different modalities are measured simultaneously in a single experiment or integrated across multiple experiments. Single-cell multimodal omics is able to reveal cell functions, discover relationships across -omes, and record dynamic biological events. Single-cell multimodal omics was named the Method of the Year 2019 by Nature Methods [62].

While single-cell technology was initially used for single-cell RNA sequencing (scRNA-seq), it quickly expanded to include other biomolecular characterizations, such as protein profiling and epigenetic analysis, and became truly single-cell multimodal omics. For example, the integration of in situ Hi-C and whole-genome bisulfate sequencing was used to simultaneously profile chromosome conformation and DNA methylome in single cells [64]. There are two types of single-cell multimodal omics approaches. The first approach measures multiple modalities one cell at a time. This approach aims to achieve comprehensive profiling via multiple modalities in each cell; however, it is restricted by the throughput and costs. The second approach can process thousands to millions of cells together by employing droplet platforms or combinatorial DNA barcoding strategies, thus achieving a high scalability and cost effectiveness [65].

### 4.5. Clinically Enriched Subtypes and Deep Phenotyping

In medical contexts, the word “phenotype” is the trait or observable characteristic of a patient in terms of his/her unique morphological, biochemical, physiological, or behavioral properties. This term is generally considered to mean changes from the normal characteristics of patients. Although phenotyping plays a key role in clinical practice and medical research, the descriptions of phenotypes in clinical notes and medical publications are often imprecise. Deep phenotyping is used to precisely and comprehensively analyze phenotypic abnormalities. By deep phenotyping, researchers can identify and describe the individual components of the phenotype.

One of the goals of precision medicine is to classify patients into subclasses that differ in phenotype, susceptibility, and/or the molecular profile of a disease or the likelihood of an adverse or positive response to a specific treatment. In order to identify those subclasses and to translate this knowledge into clinical care, it is critical to utilize computational resources to capture, store, and exchange phenotypic data and to use sophisticated algorithms to integrate the clinical phenotypic data with omics profiles, genomic variation, and other biological information [66].

The precise matching of deep genomic and phenotypic models is critical to precision medicine [67]. While the genotype of patients can be accurately determined now, phenotype information has been partial, generic, and time-consuming to gather. So far, information about individual phenotypes is far less than what we know about the genetic variations among individuals. Quite often, the current phenotypic descriptions fail to depict the nature of the disease or its stage. The problem is particularly severe regarding rare diseases due to the limited cases and the incomplete phenotypic descriptions. Phenotype descriptions are typically “sloppy or imprecise”, according to a 2012 review [66].

To overcome these difficulties, deep phenotyping is needed to exhaustively and comprehensively examine the individual components of a phenotype in a way that goes beyond what is typically recorded in medical charts. Through such deep phenotyping, details about disease manifestations are gathered in a more individual and finer-grained way. The collected data will also be integrated with other kinds of information. Deep phenotyping can provide new types of big data with more specificity, which has the potential to connect disease subtypes and genetic variations. The deeper you go, the more you know.

Existing approaches for clinical phenotyping typically cluster individuals into subtypes using electronic health record (EHR) data. In autism, for example, different subtypes were identified by using hierarchical agglomerative clustering over individuals based on *ICD-9* codes (their set of comorbid conditions). It is revealed through the subtyping that patients in some subtypes are more likely to experience auditory complications, whereas patients in other subtypes are more likely to experience seizures [68]. The main advantage of subtyping with *ICD-9* is that the data are in a standardized format and are readily available in administrative billing records. However, this type of subtyping is highly sensitive to practice patterns.

Phenotyping could also be based on disease activity trajectories. For example, four subtypes of scleroderma were identified based on lung disease activity trajectories tracked over 15 years. In addition, three distinct subtypes were identified based on joint analysis of the lung and skin trajectories, which yields subpopulations that show distinct autoantibody profiles and comorbidity patterns [24].

Through a data-driven approach based on the motor and nonmotor features of Parkinson’s disease (PD), four PD subtypes were identified: Subtype 1 was mildly affected in all domains, Subtype 2 was predominantly characterized by severe motor complications, Subtype 3 was affected mainly in nondopaminergic domains without prominent motor complications, and Subtype 4 was severely affected in all domains [69].

### 4.6. Integrative Analysis

In the upcoming future, it is likely that better subtyping will enable accurate prognoses of disease trajectories and enable treatment planning and improve prognosis. It is possible that molecular data will be linked with rich clinical data, which will significantly advance our knowledge regarding diseases and their treatment. It is also possible that molecular mechanisms underlying various disease subtypes will be discovered, which will facilitate the development of various novel biomarkers and the applications of novel treatments.

To make those things happen, the diverse data surrounding an individual’s health need to be careful integrated through integrative analysis. These data including their clinical, molecular, and environmental data. This data integration will enable us to identify naturally occurring subtypes and tailor treatments to these subgroups. Moreover, causal pathways associated with the phenotypically differentiated subgroups will also be discovered by this integrative analysis, which will facilitate the development of treatment programs appropriate to each subtype [24].

Despite significant progress in utilizing these diverse molecular and clinical datasets, much work remains. One remaining issue is the limitation of the current analytic method in the integration of diverse omics data. Although we know that these diverse omics data are interdependent, in practice, our current analytic methods tend to treat them as independent components. For example, the epigenomic process (DNA methylation) affects gene transcription (levels of mRNA expression). Thus, data modeling based on the assumption of independence is likely to be biased.

The heterogeneity of the different data types is another challenge faced by integrative analysis. For example, some data are measured repeatedly (such as blood cell counts and functional lung tests), while other data (for example, gender or DNA sequence) are measured only once. Some markers are categorical, while others are continuous. Different sources of measurement noise and bias can also affect the accuracy of the measurements. These nuisance sources of variability need to be considered when we classify meaningful subtypes. Multiresolution models that integrate diverse markers and incorporate knowledge of their biology and measurement process are therefore needed.

## 5. Tailored Treatment for the Disease Subtypes

**Figure 7:**
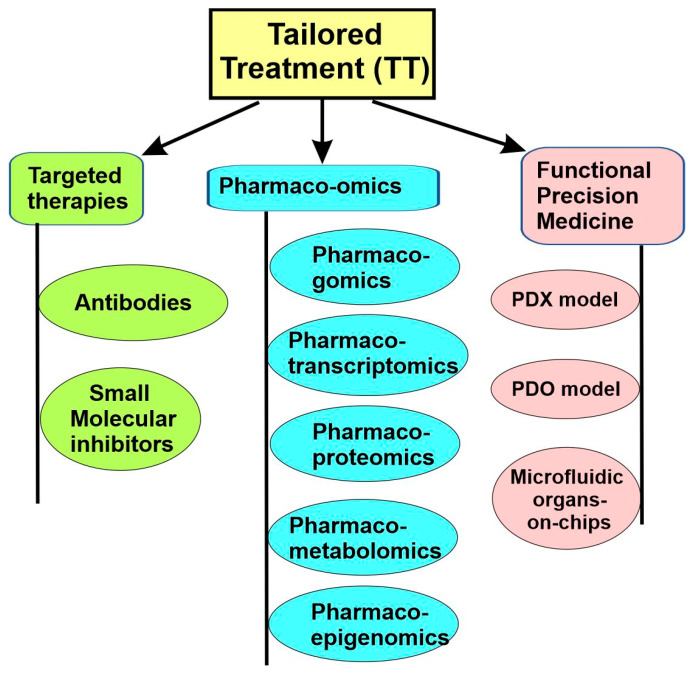
Tailored treatment (TT). TT is achieved through the following strategies and approaches. First, the development of a targeted therapy that specifically targets identified molecular subtypes. Second, pharmaco-omics to identify the right drugs and right dosages tailored to the individual based on the person’s particular genetic/molecular makeup. Third, functional precision medicine (FPE), which is based on the direct exposure of tissues derived from affected individuals to different drugs.

As we mentioned earlier, for precision medicine to be successful, an effective therapeutic treatment should be available and tailored to each disease subtype. This can be achieved through the following approaches. First, the development of a targeted therapy that specifically targets identified molecular subtypes. Second, the use of pharmaco-omics to identify drugs tailored to the individual, which selects the right drugs and right dosages for the individuals based on the person’s particular genetic/molecular makeup, as well as a person’s environment, diet, age, lifestyle, and state of health. Third, the use of functional precision medicine, in which the direct response of patient-derived cancer cells to different drugs in vitro guide which therapy should be used (Figure 7).

All three approaches have played an important part in precision medicine research. The targeted therapy approach has existed for several decades, with many diverse strategies developed. Pharmaco-omics is slightly more recent and has made significant progress in the last decade. Finally, functional precision medicine is the newest and is likely to play a significant role in the future.

### 5.1. Targeted Therapies

Targeted therapy employs specific therapeutic drugs to target specific pathogenic molecules or cells. In this case, the drug specifically binds only to pathogenic molecules or cells without affecting normal cells, thus enabling personalized and precise treatment [70]. The concept of targeted therapy was proposed by Paul Ehrlich over a century ago. Paul Ehrlich first proposed the concept of “magic bullets” in 1907 and envisioned, that just like a bullet hitting a specific target, there could be a way to specifically target and kill the invading microbes causing disease in the body, without harming the body itself [71]. Ehrlich’s genius inspired multiple ground-breaking studies that resulted in targeted therapies for treating various human diseases [72].

The completion of the Human Genome Project and the rapid advance in omics technologies allowed the precise detection of changes in the genome, transcriptome, and proteome. By using these novel technologies and enormous new data, scientists can design new drugs specifically for the disease-causing molecules by exploring the mechanisms underlying the diseases’ progression [73]. By specifically targeting the abnormal genes or proteins, the novel drugs enable effective and personalized treatment. Additionally, the recent striking breakthroughs in cell therapy and gene editing techniques have advanced targeted therapy into a vigorous development stage. The use of targeted therapy in oncology is an excellent example. So far, various drugs have been developed to specifically target different cancers with considerable effectiveness. These drugs include trastuzumab, which specifically targets HER2-positive breast cancer, and chimeric antigen receptor-modified T (CAR-T) for treating hematological malignancies [74]. Targeted therapy is also playing an important role in the treatment of other diseases, including cardiovascular diseases [70], autoimmune disease [75], cystic fibrosis [76], and many others [1].

### 5.2. Targeted Therapy for Cancers

Targeted therapy or molecular targeted therapy has been narrowly defined as a novel medical treatment for cancers due to its significant, successful, and widespread application in cancer treatment. In this section, we will review the progress of targeted therapy in cancers. However, it is important to realize that similar ideas, technologies, and approaches have also been successfully applied to other diseases.

Until the late 1990s, except for hormone treatments, nearly all drugs used in cancer treatment acted by killing cancer cells during either DNA replication or mitosis. While their primary effects were on cancer cells, these chemotherapy drugs also killed some normal cells. Since the 1980s, scientists have realized that many of the growth factors, receptors, and downstream signaling molecules of cancer cells are the products of oncogenes. Targeted therapies were thus developed to specifically target these cancer-specific mechanisms. For instance, drugs were developed to block cancer-specific growth signals, including antibodies, such as trastuzumab (Herceptin), gefitinib (Iressa), imatinib (Gleevec), and cetuximab (Erbitux). Another type of targeted therapy aims to block angiogenesis within tumors. Antiangiogenesis agents stop tumors from making the new blood vessels they need to keep growing. Judah Folkman proposed this concept in the early 1970s; however, the first angiogenesis inhibitor, bevacizumab (Avastin), was approved 30 years later in 2004. Currently used to treat advanced colorectal, kidney, and lung cancers, bevacizumab is being studied as a treatment for many other types of cancer too. Since then, many new angiogenesis inhibitors have also become available for clinical application.

In recent years, molecular drivers of cancer have increasingly become the focus of novel drug development. There are two main approaches for targeted therapy: antibodies and small molecules (Figure 7).

#### 5.2.1. Antibodies

##### Overview

Specific monoclonal antibodies (mAb’s) are the earliest developed targeted therapeutics. In general, mAb’s function with high specificity; however, their targets are often limited to the cell surface, and they can only be administrated through intravenous or subcutaneous injection due to their large molecular size.

The “Magic bullet” concept was envisioned by Ehrlich more than a hundred years ago. However, it only became practical following the development of hybridoma technology in the 1970s by Kohler and Milstein [77]. Hybridoma technology produces mAb’s highly specific to their targeted antigens. Unfortunately, because of their murine origins, the efficacy of the first-generation mAb’s were hindered by their immunogenicity and poor ability to recruit immune effectors in clinical applications [78,79]. To overcome these hurdles, chimeric and humanized mAb’s were developed. These novel antibodies retain targeting specificity by incorporating either the entire murine variable regions (chimeric antibodies) or portions of the murine variable regions (humanization) but replace the murine Fc domain with human Fc domains to reduce the immune response from the human host.

The customization of the IgG for desired functions becomes possible following the transition to human IgG backbones. Human IgG1 is the most used isotype. IgG1 mediates Fc-domain-based functions, including antibody-dependent cellular cytotoxicity (ADCC), and complements fixation effectively. Further modifications, including alterations in glycosylation that enhance ADCC or modifications in size and antigen-binding affinity that increase the ability of the mAb to penetrate solid tumors, are being made in the next generation of mAb’s currently under development.

CD20-targeting rituximab (Rituxan^®^) and HER2-targeting trastuzumab (Herceptin^®^) were the first two mAb’s approved by the FDA in 1997 and 1998, respectively. Since then, significance progress has been made. Twenty-five years later, antibody therapeutics have emerged as clinically and commercially successful pharmaceuticals with 100 FDA-approved mAb’s. Oncology is the field having the biggest success with nearly half of the currently marketed therapeutic mAb’s. Tumor antigens that have been successfully targeted include CD20, CD30, CD52, HER1, epidermal growth factor receptor (EGFR), vascular endothelial growth factor (VEGF), and cytotoxic T lymphocyte-associated antigen 4 (CTLA4). Serological, proteomic, genomic, and bioinformatic databases have also been used to identify antigens and receptors that are worth targeting. These targets include proteins overexpressed in tumor cell populations or that are linked to gene mutations identified as driving cancer cell proliferation, including EGFRvIII, MET, CTLA4, and fibroblast activation protein (FAP) [80].

**Figure 8:**
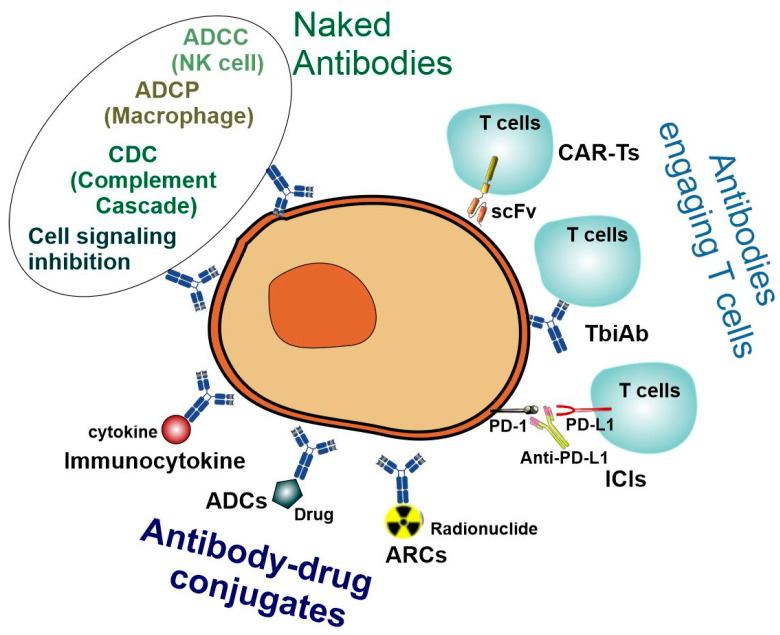
The antibody-based cancer therapies can be broken down into three categories based on their different mechanisms of action: (i) naked antibodies based on the natural properties of IgG, including ADCC, ADCP, CDC, and the inhibition of cell signaling; (ii) engineered antibodies engaging cytotoxic T cells, including immune checkpoint inhibitors (ICIs), T-cell-engaging bispecific antibody (T-biAb), and chimeric antigen receptor T cells (CAR-Ts) using single-chain variable fragment (scFv); and (iii) antibody conjugates that deliver cytotoxic payloads, including antibody–drug conjugates (ADCs), antibody–radionuclide conjugates (ARCs), and immunocytokines.

The antibody-based cancer therapies can be classified into three categories based on their different modes of action: (i) naked antibodies based on the natural properties of IgG, (ii) engineered antibodies engaging cytotoxic T cells, and (iii) antibody–drug conjugates that deliver cytotoxic payloads (Figure 8) [81,82,83].

##### Therapeutic Antibodies Based on Natural Properties

Initially, the development of mAb’s as therapeutics was based on their natural properties. mAb’s naturally have a high affinity and specificity to virtually all their antigen targets. They are able to block receptor–ligand interactions. They have a long circulatory half-life, and they engage the proteins and cells of the innate immune system to mediate complement-dependent cytotoxicity (CDC), ADCC, and antibody-dependent cellular phagocytosis (ADCP) [81,84] (Figure 8).

##### Antibody–Drug Conjugates and Antibody–Radionuclide Conjugates

Despite the early success of the mAb’s based on natural properties, it was realized that treatment using mAb’s alone is often insufficient and less lethal against cancer cells compared to chemotherapy. To solve these problems, a novel concept, known as the antibody–drug conjugate (ADC), was developed to bridge the gap between mAb’s and cytotoxic drugs for an improved therapeutic efficacy. The ADC consists of a tumor-targeting mAb conjugated to a cytotoxic payload through a chemical linker, combining the precise targeting of the mAb with the potent effectiveness of the cytotoxic drug. Moreover, because the antibody is typically large and hydrophilic, the antigen-independent uptake of the cytotoxic payload in normal, antigen-negative cells is limited, reducing unspecific targeting and increasing the therapeutic index [85,86]. A successful example is the treatment of HER2-low metastatic breast cancer with trastuzumab deruxtecan (DS-8201), a conjugate composed of an anti-HER2 antibody, a cleavable tetrapeptide-based linker, and a cytotoxic topoisomerase I inhibitor [87,88]. HER2-low metastatic breast cancer patients are not responsive to trastuzumab, which poses a big challenge to treat this group of patients. A series of clinical trials from phase I to phase 3 revealed that trastuzumab deruxtecan treatment resulted in significantly longer progression-free and overall survival than the physician’s choice of chemotherapy [87,88].

Since the first ADC, Mylotarg^®^ (gemtuzumab ozogamicin), was approved in 2000 by the US Food and Drug Administration (FDA) [89], 14 additional ADCs have received market approval. Most encouragingly, over 100 ADC candidates are currently in the various clinical stages of testing for future use. This kind of anticancer drug, known as “biological missiles”, is leading a new era of targeted cancer therapy [86] (Figure 8).

Antibody–radionuclide conjugates (ARCs) are another type of antibody conjugate used to deliver cytotoxic payloads to cancerous cells. Because cancer frequently presents as a disseminated disease, it is critical to deliver cytotoxic radiation to both the primary tumor and distant metastases with reduced exposure to normal tissues. ARCs were designed to achieve these objectives and have been in development for many years as an anticancer strategy. When delivered at a sufficient dose and frequency to a neoplastic mass, radiation can kill tumor cells [90,91]. So far, there are only two FDA-approved ARCs: tositumomab-I131 (Bexxar^®^) and ibritumomab tiuxetan (Zevalin^®^). Both ARCs were approved in the early 2000s, and both are anti-CD20 mAb’s conjugated to β-emitting radionuclides (90Y and 131I, respectively) [92] (Figure 8).

##### Engineered Antibodies Targeting Cytotoxic T Cells

The most recent and promising advancement in antibody therapy is the development of engineered antibodies targeting cytotoxic T cells. The natural properties of antibodies are confined to deploying the innate immune system. However, following the realization that cancer and the immune system are in a constant battle involving immunoediting, immunosuppression, and immunosurveillance [93], novel research has focused on engaging components of the adaptive immune system, and, in particular, cytotoxic T cells [81]. The first such FDA-approved antibody, Ipilimumab, accomplishes this by inhibiting CTLA-4, a protein receptor that normally downregulates the immune system [94]. So far, the FDA has approved 13 engineered antibodies targeting T cells, including 7 immune checkpoint inhibitors (ICIs), one T-cell-engaging bispecific antibody (T-biAb), and 5 chimeric antigen receptor T cells (CAR-Ts) (Figure 8).

These ICIs have broad applications in solid tumor treatment and act by suppressing the cancer cell and antigen-presenting cell (APC)-mediated inhibition of tumor-infiltrating cytotoxic T cells [81]. These ICIs either target cancer cells and APCs in the tumor microenvironment via PDL1 (and durvalumab (Imfinzi^®^), avelumab (Bavencio^®^), and atezolizumab (Tecentriq)) or target T cells via CTLA4 (ipilimumab (Yervoy^®^)) or PD1 (cemiplimab-rwlc (Libtayo), nivolumab (Opdivo^®^), and pembrolizumab (Keytruda^®^)) [95]. Currently, many ICIs are in clinical trials, including 13 antibodies to first-generation targets CTLA4, PD1, and PDL1, as well as 4 antibodies to next-generation targets LAG3, NKG2A, TIGIT, and TIM3.

The newest progress in the field includes the development of biAb’s that simultaneously engage two different immune checkpoints, such as PD1 × LAG3, PD1 × CTLA4, and PDL1 × CTLA4. Combining two ICIs in one biAb as opposed to a mixture of two individual mAb’s has cost-saving potential.

#### 5.2.2. Small Molecule Inhibitors

Small molecules can potentially bind to a wider range of intracellular and extracellular targets by virtue of their small size. To date, the FDA has approved 43 small molecule inhibitors for oncology applications, mostly based on the prolonged survival of patients with advanced cancer that were refractory to conventional chemotherapy. Most of these approved small molecule inhibitors function to inhibit the activities of intracellular kinases. Moreover, many of these small molecule inhibitors have shown superiority over cytotoxic chemotherapy, with fewer side effects as a first-line therapy in the recurrent or metastatic setting.

In general, there are two types of small molecule inhibitors, selective small molecule inhibitors and multikinase inhibitors, based on their selectivity. Selective small molecule inhibitors only bind to and inhibit fewer targets and, in some cases, only a single component of cell signaling. The presence or absence of specific predictive biomarkers in the tumor or blood samples are used as the basis to select the patients for the treatment with this type of small molecule inhibitor. In contrast, multikinase inhibitors have a low specificity and exert their anticancer activity by simultaneously targeting a broad range of human kinases. The use of these drugs is generally based on histological diagnosis, without the need for additional individualized patient selection [73].

### 5.3. Pharmaco-Omics

Pharmaco-omics is a field of research that uses “omics” sciences to provide insight into molecular mechanisms involved in individual variations in drug response phenotypes. Pharmaco-omics can reveal mechanisms of drug responses and guide the application of various drugs [53,96]. Pharmacogenomics is the earliest and most developed branch in pharmaco-omics. Other relatively newer and developing branches include pharmacotranscriptomics, pharmacoepigenetics, pharmacoproteomics, pharmacometabolomics, and pharmacomicrobiomics (Figure 7).

#### 5.3.1. Pharmacogenomics

Pharmacogenetics and pharmacogenomics investigate the effects of genetic variations on drug responses and dispositions. Genetic variation may occur in single genes or in multiple genes (polygenetic). If the frequency of a genetic variation is more than 1% of the population, it is defined as a genetic polymorphism [53,97]. Originally, pharmacogenetic studies focused on monogenic variations that are involved in drug transportation and metabolism. The goal of pharmacogenetics is to make an informed and best choice of medical treatment based on a patient’s individual genetic information. However, with advances in molecular pharmacology and human genomics in the late twentieth century [98,99], pharmacogenetics evolved into the more comprehensive pharmacogenomics.

Pharmacogenomics examines the effect of genetic variation on drug responses in the context of the entire genome, rather than just a single gene. These genes can influence both pharmacokinetics, factors that affect the concentration of a drug that reaches the target, and pharmacodynamics, the effect of the drug itself. This genetic variation is analyzed among large groups of people within the population, which can, for instance, be useful to see how different drugs affect different ethnic groups. Pharmacogenomics is also increasingly moving across the “translational interface” into the clinic and is being incorporated into the drug development process.

Both pharmacogenetics and pharmacogenomics support precision medicine by identifying drugs tailored to the individual’s particular genetic makeup. Although a person’s environment, state of health, lifestyle, age, and diet can also influence that person’s response to medicines, an individual’s genetic makeup is the key to selecting personalized drugs that work best for the patients with fewer side effects.

The earliest examples of pharmacogenetics were reported in the 1950s and 1960s. At the time, large differences among patients in their response to “standard” drug doses were observed. These differences are usually coupled with individual variations in the plasma or urinary concentrations of drug or drug metabolites. The two best examples are the genetic variation in the enzymatic acetylation of drugs, such as the antituberculosis drug isoniazid [100], and the genetic variations in butyrylcholinesterase (BCHE), an enzyme that hydrolyzes the short-acting muscle relaxant [101,102]. Both enzymatic activities involved pharmacokinetic variation and behaved as monogenic traits. Some patients experienced prolonged muscle paralysis, a serious and potentially lethal adverse side effects following the treatment with succinylcholine [103]. It was revealed that this was caused by the inheritance of an “atypical” form of BCHE [101,102]. Later studies found that the BCHE allele encoding the most common atypical form of the enzyme is the substitution of A209 by G, which results in the change of Asp70 to Gly [104,105].

A more recent example of pharmacogenomics is the study of the anticoagulant warfarin. Warfarin pharmacogenomics takes us beyond monogenic traits into polygenic variation in both pharmacokinetics and pharmacodynamics, which represents a substantial component of the discipline’s future [97].

Warfarin is the most widely prescribed oral anticoagulant drug in both North America and Europe [106]. However, adverse reactions, including both undesired coagulation and hemorrhage, are very serious and continue to complicate warfarin therapy. It was found that some observed variation in dosage selection and side effects was due to two common CYP2C9 polymorphisms [107]. However, this was not enough to fully explain the variance in the end-dose of warfarin in patients given the same drug amount [108]. The answer was only found in later pharmacodynamic and pharmacogenomic studies in 2004. These showed that the gene encoding the target of warfarin, vitamin K epoxide reductase complex 1 or VKORC1, also played a key role, and only a combination of VKORCI haplotyping and CYP2CP genotyping fully explained the variance in warfarin dose [106]. When both VKORC1 haplotypes and CYP2C9 polymorphisms were determined, it was possible to assess genetic variation both in the drug target and in the drug metabolism, which allows the move beyond monogenic pharmacogenetics. This example represents the polygenic model that many investigators expect to observe with increasing frequency in the future.

With the conclusion of the Human Genome Project, the focus has shifted from individual genes to the entire genome. The promise of pharmacogenomics is high as it could apply to every individual taking any medication.

#### 5.3.2. Pharmacotranscriptomics

Pharmacotranscriptomics is still a very new field of research that has just begun to flourish; however, it holds promise to inform biomarker development, enable target discovery, and evaluate drug efficacy beyond pharmacogenomics [109]. Pharmacotranscriptomics is based on individualizing drug treatment and dosing based on interindividual transcriptome variations [53]. Recent examples include the pharmacotranscriptomic profiling of resistant triple-negative breast cancer cells treated with lapatinib and berberine [110] and the pharmacotranscriptomic analysis of gene networks regulating ferroptosis in cancer [111].

#### 5.3.3. Pharmacoepigenetics

Pharmacoepigenetics is the study of the epigenetic basis for individual variation in drug responses [112,113]. It is known that the expressions of drug metabolizing enzymes, drug transporters, and nuclear receptors are all regulated by epigenetic modification, which affects drug responses, adverse effects, and resistance. Some approved epigenetic-focused therapies act by modulating acetylation or methylation genes [53]. One study indicated that statin therapy is associated with epigenetic modifications in individuals with Type 2 diabetes [114]. A recent study focused ona genome-wide methylation analysis of responsiveness to four classes of antihypertensive drugs using a double-blind crossover study. The findings from this study indicated that ACY3 genetic and epigenetic variation play important roles in the blood pressure response to bisoprolol [115].

#### 5.3.4. Pharmacoproteomics

Pharmacoproteomics is a branch of proteomics. It applies proteomics to discover novel drugs and assess drug effects. It is the most promising and rapidly evolving branch of proteomics in the post-genomic era. Pharmacoproteomics has inherited all the promises that pharmacogenomics has left unfulfilled [53,116]. Similarly to what was discussed above regarding the difference between proteomics and genomics/transcriptomics, pharmacoproteomics can reveal the patient-to-patient variation more accurately than pharmacogenomics/pharmacotranscriptomics. The proteins are what carry out the biological functions and many genetic changes may not actually translate into changes in the protein milieu. Interindividual variations may be quantitative and qualitative and may also be altered in diseases and after drug administration. A recent study provided a good example by using a pharmacoproteomics approach to identify potential molecular targets associated with kidney preservation injury. It revealed that specific metabolic enzymes are involved in preservation injury and in the mechanism underlying the doxycycline protection of the kidney during cold perfusion [117].

#### 5.3.5. Pharmacometabolomics

Pharmacometabolomics focuses on the interactions between pharmacology and the patient’s metabolic phenotype [118]. Pharmacometabolomics uses a metabolomic approach to understand the pharmacokinetics and pharmacodynamics of the drug; to identify novel targets for drug research, to discover biomarkers relevant to the response to a drug, and to determine the factors that can alter drug metabolism [118,119]. A good example is the research that identified N Methyl glycerine as a novel target for treating prostate carcinoma [120] and 2-hydroxyglutarate for acute myeloid leukaemia [121]. Recently, pharmacometabolomics was used to study the inhaled corticosteroid response in individuals with asthma [122]. Raman-guided subcellular pharmacometabolomics was also recently explored as a noninvasive method to study metabolites within single live cells [123].

### 5.4. Functional Precision Medicine

#### 5.4.1. Overview

Functional precision medicine (FPM) is an approach based on the direct exposure of tissues derived from affected individuals to drugs [124]. Functional precision medicine emerged to complement the shortfalls of omics-based precision medicine. Despite many successful stories, the clinical impact of a precision medicine approach has not reached the level that was expected. One of the problems is that most precision medicine approaches rely on genomics and genomics does not automatically predict the response to a given treatment. Indeed, multiple factors, including the tumor microenvironment, protein modifications, gene expression, the activation of alternative molecular pathways, and microbiota, can influence the response of the individual patient to the treatments. These factors contribute to interpatient heterogeneity, even among individuals with the same genomic alterations. This heterogeneity is certainly the epiphenomenon of the various mechanisms contributing to the variations that are both inter- and intratumoral.

In contrast to the static approach of existing omics-based precision medicine, FPM generates functional and dynamic data. These data may encompass key vulnerabilities driven by genomic aberrancies, epigenetic modifications, and/or signaling pathway alterations. The FPM models derived from patients have been powered by increases in the heterogeneity of human cancers, the rapid development of novel technologies, and the availability of novel cancer drugs.

Although the current FPM concept is new, similar approaches, such as growing cancer cells in mouse models or in vitro cell cultures for chemosensitivity testing, began more than half a century ago. In fact, nearly every curative cytotoxic regimen used today in oncology was derived from information generated by these early studies [125]. FPM has resulted in dramatic increases in the number of available drugs, novel technology, and biological knowledge, which, in turn, has re-generated interest in the ex vivo analysis of tumor cells to guide therapy.

The development and validation of short-term or long-term models is a key aspect of FPM. A successful model should retain fidelity to the human tumors from which it arises. The current pre-clinical models include patient-derived xenografts (PDXs), patient-derived organoids (PDOs), and microfluidic organs-on-chips (Figure 7).

#### 5.4.2. Patient-Derived Xenograft (PDX) Models

PDX models have been developed for many different cancers, and they are rapidly replacing traditional cell lines as preferred models for performing basic and translational research in precision medicine.

Drug development using animal models was first reported for leukemia in 1950 [126]. Since then, many types of murine models transplanted with human tumors have been developed to study responses to chemotherapy. Many of these models were generated in immunodeficient mice. Typically, tumor cell lines that had been propagated in cultures were injected into mice to allow the development of tumors in mice.

However, tumor cell lines are very different from the primary tumor following the long-time culture in the laboratory. They frequently acquire unanticipated phenotypes [127]. To overcome the limitation, PDXs have been established as a model more likely to mimic human tumors, which makes them a useful tool for translational research [128,129]. The implantation of small pieces of tumors surgically dissected from cancer patients into highly immunodeficient mice allows tumor growth and a subsequent transplantation into secondary recipient mice. PDXs possess server advantages, including the maintenance of the cellular and histopathological structures of the original tumors as well as the genomic and gene expression profiles of the original tumors. Accumulated data suggest that the sensitivity to standard chemotherapeutics in PDXs closely correlates with clinical data for patients from which the PDXs are derived [127,130]. The PDX has been used for many different cancers, including colon adenocarcinoma [1,131], pancreatic cancer [132,133], cholangiocarcinoma [134], glioblastoma [135], glioma [136], medulloblastoma [137], breast carcinoma [130,138,139], lung cancer [140], malignant melanoma [141], head and neck squamous cell carcinoma (HNSCC) [142], ovarian cancer [143,144], and bladder cancer [145].

While the PDX models have made significant contributions to cancer research and treatment, several issues still need to be addressed to improve their utility for functional precision medicine. These include (1) optimizing the implantation site, (2) reducing the time needed for PDX tumor tissue generation, (3) increasing the successful rate of engraftment, (4) managing the replacement of human stroma with murine stroma, (5) evaluating the contribution of the host immune system to responses, and (6) overcoming the problems associated with Matrigel [127].

#### 5.4.3. Patient-Derived Organoids (PDOs)

Due to the difficulty of long-term in vitro cultures of patient-derived cells/tissues, functional precision medicine has been technically impossible for many years. This has completely changed with the development of a new primary culture technique: organoids.

The identification of the Lgr5+ adult stem cell population within the intestinal crypt [144] and the subsequent development of the growth factor recipe for human intestinal organoids [145] made the development of organoids possible and opened the way to the generation of organoids from many tissues, including the colon [146,147,148], brain [149], prostate [150], pancreas [151], liver [152], breast [51,130], stomach [153], esophagus [154], endometrium [26,155], lung [156], and the head and neck [151], which led to the development of “living biobanks” of organoids from many healthy and diseased issues.

**Figure 9:**
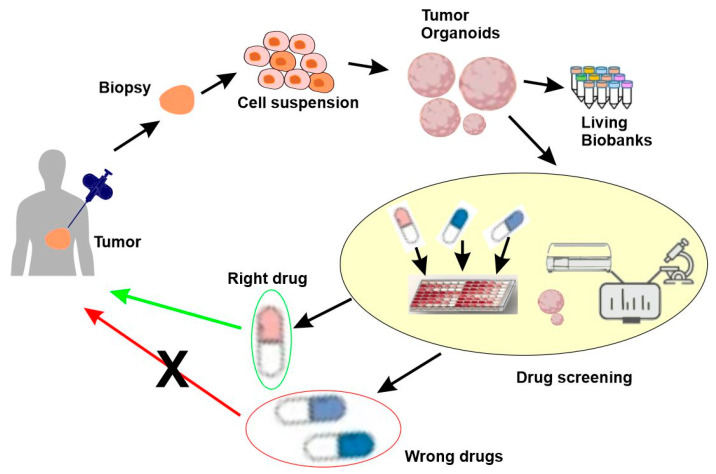
Patient-derived organoids (PDOs) for individualized tumor response testing. Tissue from a patient’s tumor is obtained through diagnostic biopsy and is then enzymatically and mechanically dissociated into a single-cell suspension. It is then embedded in a basement membrane extract drop and grown in specialized culture medium to form organoids. Following the confirmation of the tumor histology, the formed organoids will be deposited into living biobanks and be used for drug screens. Various read-outs can be obtained to define the PDO drug screen response (including organoid size, viability, and coculture cytokine measurements). Effective drugs identified through the screening are then used to treat the patient.

For individualized tumor response testing, tissue from a patient’s tumor is obtained through diagnostic biopsy. The tissue specimen is dissociated enzymatically and mechanically into a single-cell suspension. The cells are then embedded in a basement membrane extract drop and grown in a specialized culture medium to form organoids. Following the confirmation of the tumor histology, the formed organoids are then deposited into living biobanks to be used for drug screens. Various read-outs can be obtained to define the PDO drug screen response (including the organoid size, viability, and coculture cytokine measurements). Effective drugs identified through the screening can be used to treat the patient (Figure 9).

The implementation of PDOs is critical for functional precision medicine. PDOs spare nonresponding patients from side effects for drugs that are already in the clinic or at an advanced development stage. It provides a platform for screening new drugs in models that are a faithful replication of the tumor. Moreover, it enables the functional validation of emerging genomic and nongenomic biomarkers. Furthermore, many studies have demonstrated that organoids could replicate the response to pharmacological treatments. In a recent study regarding colon cancer, an outstanding 100% sensitivity, 93% specificity, 88% positive predictive value, and 100% negative predictive value were achieved using organoids [147].

Ultimately, this technology is still in its infancy. For example, while all studies reporting on the genomic profile show that PDOs replicate both the mutation and copy number profile, only a few studies examined transcriptomic similarities and none of them discussed proteomics [157]. However, current PDOs are not sophisticated enough to assess the effect of the microenvironment of the tumor, and the lack of standardized protocols and quality controls also hinders the development and application of PDOs.

#### 5.4.4. Microfluidic Organs-on-Chips

Organ chips are microfluidic cell culture devices composed of optically clear plastic, glass, or flexible polymers that contain perfused hollow microchannels populated by living cells arranged to recreate tissue- and organ-level structures and functions in vitro, which simulates in vivo tissue- and organ-level physiology and pathophysiology [158,159]. By recapitulating the multicellular architectures, vascular perfusion of the body, and physicochemical microenvironments, these devices overcome the major shortcomings of conventional 2D or 3D culture systems and produce much higher levels of tissue and organ functionality. Moreover, organ chips also enable high-resolution and real-time imaging and thus in vitro analysis of genetic and biochemical activities of living cells in a functional tissue and organ context.

One drawback of normal 3D culture systems is the difficulty of carrying out high-resolution imaging and determining where in the tissue to look. This is even more difficult when the functionality and complexity of the cultured cells increase in a way similar to visualizing processes in living organs. In contrast, in organs-on-chips, cell types of one tissue type can be positioned precisely and consistently relative to those of other tissue types in organs-on-chips. Organs-on-chips make it straightforward to integrate cell cultures with analytical assays like multiple electrode arrays, microfluorimetry, and fluorescence confocal microscopy, among others. In the future, molecular reporters and nanoscale sensors could be incorporated, which will allow the assessment of multiple linked organs-on-chips using multiplexed microscopes and robotic systems Furthermore, the organs-on-chips model can control the fluid flow to enhance the function and long-term survival of many cell types. A good example is the long-term culturing of human lung cells [160].

Unfortunately, there are also some weaknesses with the organs-on-chips model. As it stands now, the technology cannot model some functions of macroscale architecture, such as the cognition in the brain and mechanical function in bones, ligaments, and tendons. The capacity and function of the microfluidic organs-on-chips are also limited by the technical challenges. In addition, comparing them to other 3D culture systems, organs-on-chips are more difficult to operate and usually have a lower throughput [161,162].

However, despite these limitations, the organs-on-chips model has great potential to advance functional precision medicine by facilitating the studies of tissue development, organ physiology, and disease etiology, as well as drug discovery and development [158,159,161,162].

## 6. Other Aspects of Precision Medicine

### 6.1. Environmental, Social, and Behavioral Factors

It is well recognized that there are many nonbiological factors that contribute to human health. One estimate suggested that only 30% of the health of an individual is determined by genetic factors; the other 70% is determined by four other factors that include human behavior (40%), social circumstances (15%), healthcare (10%), and environmental exposures (5%) [163]. Thus, precision medicine must address disease prevention and treatment in a manner that considers individual differences in environments and lifestyles in addition to genetics.

Despite the excitement surrounding precision medicine, there are criticisms that 20 years of burgeoning precision medicine research has not had a measurable effect on population morbidity or mortality and has in fact drawn research support away from other matters of public health importance [164]. Some researchers argue that one reason for this is that not enough attention and resources have been given to incorporate environmental and social factors into precision medicine [165].

Many negative health impacts of a poor environment can be mitigated with proper lifestyle changes. A better understanding of the genetic predisposition to disease can guide these changes [166]. Therefore, only by incorporating both environmental and genetic factors can precision medicine reach its full potential. It can accomplish this with a robust prediction model that can accommodate complex computational equations to integrate all these determinants. While this model is easier to accomplish for diseases with monogenic inheritance, like cystic fibrosis, it is difficult to build for multifactorial diseases like Type 2 diabetes mellitus and coronary artery diseases, which account for 71% of global mortality in the modern world. We are still in the process of improving our understanding of the interactions between various omics and social/environmental factors to develop more sophisticated predictive models. There is hope that this could be achieved in the near future in light of progress in various omics, digital health, and big data analytics. This next-generation predictive model will hopefully be able to predict the personalized risk scores of any given individual for any disease, which would have immense applications in preventive, diagnostic, therapeutics, and prognostic domains [53].

### 6.2. Electronic Health Records

An electronic health record (EHR) is an electronically stored digital version of a patient’s paper chart. It contains a patient’s medical history, immunization dates, diagnoses and test results, treatment plans, and medication [167]. The information recorded in EHRs is a combination of structured and unstructured data. Precision medicine has largely been enabled by the integration of genomic and other data with EHRs [168].

The development of EHR was made possible by the new computer technology developed in the 1970s. Early EHRs were developed between 1971 and 1992 for the purpose of billing and scheduling. Later, EHRs were developed as a clinical system to help improve medical care and for use in medical research. Now, EHR records are being created, used, edited, and viewed by multiple independent entities, including primary care physicians, hospitals, insurance companies, and patients. EER has changed the dynamics of the patient–doctor relationship through things like clinician–patient emails, virtual consults, and telemedicine [169].

As EHRs are gradually adapted to empower precision medicine implementation, they also increasingly enable new paradigms of genomic discovery and biomedical research embedded in health systems. Personalized medicine implementation will increasingly rely on EHRs to store and integrate relevant genomic information into clinical care. However, some issues remain to be solved. As the EHR is not designed for collecting data for research, the deficiencies like data inaccuracy, missing data, and bias pose challenges for using EHRs for research.

Special ethical and scientific considerations should be taken into account when linking genomic data with EHRs. In addition, many developing countries lack the robust healthcare and information technology infrastructure to broadly implement EHRs [167,170].

### 6.3. Digital Health

Simply put, digital health is the use of information and communication technologies for health [171,172]. Digital health technologies (DHTs) include both hardware and software solutions and services, such as EHRs, telemedicine, mobile health, clinical decision support systems (CDSSs), electronic prescribing systems, computerized physician order entry, wearable devices, and web-based health services. Digital health enhances the efficiency of healthcare delivery, facilitates the understanding of health challenges faced by people, and makes medicine more precise and personalized [172,173]. Therefore, digital health is a potential aspect of precision medicine in which patients can participate in partnership with clinicians and researchers. One way for patient to participate is to collect data by using FDA-approved mobile devices or apps, such as a mobile heart monitor app or a continuous glucose monitor. Such data can provide a physician with immediate information about a patient. When collected from large numbers of people, this information can reveal clinically useful patterns and trends. Patients will also be more motivated by participating in the data collection process. They could be more willing to recognize changes in their health, to track and adjust their behavior, and to follow care plans.

The creation of an integrated system that combines digital health, omics data, and environmental data is necessary for precision medicine development.

### 6.4. Big Data Analytics and Artificial Intelligence

Big data are the combination of all the data obtained from complex biomolecular assays, clinical investigations, and social and environmental factor evaluations for an individual and subpopulation [174]. Analysis of big data is essential for the success of precision medicine [53].

Due to their volume and variety, big data in healthcare and medicine are difficult to analyze and manage with traditional software or hardware [175]. Big data analytics covers the integration of heterogeneous data, data quality control, analysis, modeling, interpretation, and validation [23]. The application of big data analytics is critically important in revealing comprehensive knowledge buried in those huge amounts of data [174]. Precision medicine not only requires data collection, management, and analysis but also data storage, sharing, privacy, mining, visualization, and integration. Because of rapid advances in biotechnologies, highly complex biomedical data are becoming available in huge volumes. To make sense of these heterogeneous data, big data analytics, including data analysis, quality control, modeling, validation, and interpretation, is important to cover application areas, such as sensor informatics, bioinformatics, imaging informatics, and health informatics [23]. In particular, big data analytics in medicine and healthcare enables the analysis of large datasets from thousands of patients, the identification of clusters and correlations between datasets, and the development of predictive models using data mining techniques [53].

Artificial intelligence (AI) is a set of technologies that allow machines and computers to simulate human intelligence at various levels. Both deep learning and machine learning are parts of AI. Machine learning allows systems to learn from data at the most basic level. Deep learning uses more complex structures to build models.

AI technologies have been developed to analyze a diverse array of health data, including multiomics data, clinical data, behavioral data, environmental data, drug data, and data from the biomedical literature. AI can process large volumes of data in a timely manner through its bioinformatics system. Thus, AI has the capacity to integrate and convert big data into useful diagnostic and therapeutic interventions [53,176].

## 7. Conclusions

The past decade has witnessed a rapid acceleration in our understanding of the genetic basis of many diseases, which makes it possible to re-define diseases at a higher resolution and, consequently, create more precise therapy. This is the goal of precision medicine, which has become a term that symbolizes the most advanced modern and future medicine.

For precision medicine to work, two essential objectives need to be achieved. First, diseases need to be classified into various subtypes. Second, targeted therapies must be available for each specific disease subtype. Disease subtyping has evolved from pure clinical phenotyping to molecular subtyping and to finally a comprehensive subtyping that combines molecular data from omics with clinical data from the HER. Tailored treatment and preventative options for various disease subtypes have also greatly expanded thanks to the contribution of targeted therapies, pharmaco-omics, and functional precision medicine.

To realize the full promise of precision medicine, a further integration of multiple levels of information is needed. This includes the integration of information at the molecular level (i.e., genome, epigenome, proteome, metabolome), clinical and laboratory data, and environmental factors. Big data analytics and artificial intelligence will be the key to accomplishing this integration.
